# Red‐backed shrike (*Lanius collurio*) versus common cuckoo (*Cuculus canorus*): An example of ineffective cuckoo–hawk mimicry

**DOI:** 10.1002/ece3.9664

**Published:** 2022-12-23

**Authors:** Ladislava Krausová, Petr Veselý, Michaela Syrová, Kateřina Antonová, Ondřej Fišer, Vanda Chlumská, Markéta Pátková, Šimon Pužej, Roman Fuchs

**Affiliations:** ^1^ Faculty of Science University of South Bohemia in České Budějovice České Budějovice Czech Republic; ^2^ Faculty of Science Charles University Prague Czech Republic

**Keywords:** brood parasitism, cuckoo–hawk mimicry, nest defense, red‐backed shrike

## Abstract

The red‐backed shrike (*Lanius collurio*) used to be one of the most common hosts of the common cuckoo (*Cuculus canorus*). Nevertheless, during the last 30 years, there is increasing evidence from Central Europe that the occurrence of cuckoo chicks in shrike nests has become scarcer, and that in some locations they have disappeared completely. Multiple hypotheses have been suggested to explain this abandonment. Here, we test the hypothesis that shrikes vigorously attack adult cuckoos, potentially resulting in ineffective parasitism. Adult common cuckoos resemble in appearance the Eurasian sparrowhawk (*Accipiter nisus*), a common predator of small passerines. One hypothesis presumes that the cuckoo has evolved this mimicry to avoid attack by small passerines when searching for their nests. Our results show that shrikes defending their nests attacked cuckoos very vigorously, more often, and more intensively than they did sparrowhawks. In the presence of a sparrowhawk dummy, parent shrikes only produced alarm calls and flew over the dummy. This suggests that cuckoo–hawk mimicry is ineffective in the case of shrikes and that they attack them much more often than they do any other presented intruder. Therefore, this activity could possibly result in the abandonment of shrikes as potential hosts for cuckoos.

## Graphical abstract

For the first time, we experimentally tested reaction of Czech red‐backed shrikes to the common cuckoo (nest parasite) and compared it with the reaction to the similarly looking Eurasian sparrowhawk (predator of adults) and the turtle dove (harmless intruder). As the shrikes are abandoned by cuckoo for 30 years in the Czech Republic, this topic can describe evolutionary changes in the relationship between parasite and host that can occur in a relatively short period of time. Despite absent evolution pressure, shrikes are still able to successfully discriminate between nest parasite—the common cuckoo, and similarly looking harmful predator of adults—the Eurasian sparrowhawk.

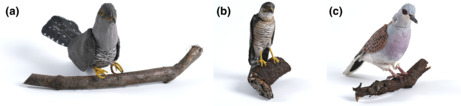

## 1 INTRODUCTION

In Europe, the common cuckoo (*Cuculus canorus*) is the most common and most studied brood parasite (Birkhead et al., 2011; Brooke & Davies, 1987; Esposito et al., 2022; Moksnes et al., 1991). The parasitic strategy of the cuckoo is very complex, with arms races developed with multiple host species. The parasitic event itself is very fast, cryptic, and planned in the period before incubation and after the first host eggs have been laid (Hamilton et al., 1965; Payne et al., 2005; Mann, 2017). The newly hatched cuckoo chick either removes all of the host eggs from the nest or, if it fails to do so, kills any of the host chicks that hatch. Therefore, the hosts have developed counter‐adaptations to prevent parasitism (Payne, 1977; Davies & Brooke, 1989; Lovászi & Moskát, 2004). The most common counter‐adaptation includes the ability to recognize the parasitic egg, but hosts may also rely on nest and egg crypsis (Feeney et al., 2014; Moskát & Hauber, 2007; Øien et al., 1996). When the adult cuckoo appears in the vicinity of the potential host nest, the host parents usually increase their vocalization, and sometimes may even attack the cuckoo physically, which is intended to chase the cuckoo away (Goławski & Mitrus, 2008; Montgomerie & Weatherhead, 1988; Spottiswood et al., 2012; Polak, 2013; Welbergen & Davies, 2008). In such a case, however, the cuckoo may rather respond by searching for the host nest (Davies & Welbergen, 2008).

The red‐backed shrike (*Lanius collurio*) is one of the species that defends its nest very aggressively, even making physical attacks on intruders (Němec & Fuchs, 2014; Strnadová et al., 2018; Tryjanowski & Goławski, 2004). In addition to aggressive attacks, shrikes fly over intruders, attempting to chase them away, and produce several different alarm calls. Ash (1970) and later Harris and Franklin (2000) described calls used when an intruder occurs in the territory of shrikes (the so‐called “chack” call, also recorded in our study) and another call produced when the intruder is attacked. Generally, the nest defense strategy of the red‐backed shrike is very effective against most of the potentially threatening species (but compared to Veselý et al., 2022).

The red‐backed shrike used to be one of the most common cuckoo hosts in Europe, but since the 1960s, the occurrence of parasitism has decreased (Lovászi & Moskát, 2004; Takasu, 2003). It is likely that the cuckoos specialized to parasite the red‐backed shrikes did not extinct, they could just shift to another host with similarly colored eggs (e.g., Sylvia warblers), as described by Moksnes et al. (2008). There are multiple theories explaining decrease in the red‐backed shrike as a cuckoo host. Lovászi and Moskát (2004) suggest the high ability of shrike populations in Hungary to recognize parasitic eggs. Adamík et al. (2009) suggest the low breeding density of shrikes in the Czech Republic resulted in the abandonment of this species by cuckoos. Another possibility is that the effective nest defense of shrikes, including high levels of aggression toward adult cuckoos, may have discouraged them from parasiting (as shown in reed warblers, Dyrcz & Hałupka, 2006).

The common cuckoo is known for its specific coloration. It is supposed that the visual appearance of the adult cuckoo mimics the Eurasian sparrowhawk (*Accipiter nisus*), a very common predator of small passerines (Bujoczek & Ciach, 2009; Götmark, 1996; Trnka et al., 2015; Trnka & Prokop, 2012). Davies and Welbergen (2008) showed that two species of tit (*Parus major* and *Cyanistes caeruleus*) cannot distinguish between cuckoo and sparrowhawk, in contrast, reed warblers (*Acrocephalus scirpaceus*), as well as great reed warblers (*Acrocephalus arundinaceus*), were able to respond differently (Trnka & Grim, 2013; Trnka & Prokop, 2012; Welbergen & Davies, 2008). The reason for this discrepancy in response may be the co‐evolution of these passerine species with the cuckoo, as neither tit species usually acts as a cuckoo host, while both warbler species commonly do.

In this study, we tested the hypothesis that red‐backed shrikes are able to differentiate between adult cuckoos and adult sparrowhawks when occurring at their nests. We also compared the responses to both of these species with responses to a harmless turtle dove (*Streptoptelia turtur*). We, therefore, decided to observe the level of aggression of red‐backed shrikes toward adult cuckoos in a situation when there is low parasitic pressure. We hypothesized that shrikes are able to differentiate among the nest parasite, the predator of adults, and the harmless control and respond to them appropriately. Moreover, we tested if the reactions of the shrikes to particular species differ in two phases of nesting—the egg‐laying phase, when the likelihood of nest parasitism is higher, and during the incubation phase, when the clutch is completed, and the threat of parasitism is lower. We expected that shrike parents would react more intensively to a brood parasite during the egg‐laying phase (Campobello & Sealy, 2010; Gill & Sealy, 1996; Grim, 2005).

## 2 MATERIALS AND METHODS

### 2.1 Study area

The study took place in the Doupov Mountains, near the town of Karlovy Vary on the southern border of a military training area (50°10′N, 13°9′E) in the Czech Republic. The main habitat is meadows or pastures with many shrubs. The study area reaches quite high densities of red‐backed shrikes nesting pairs (up to 18 pairs per km^2^; Němec, personal observation) and with no evidence of cuckoo parasitism for the last 20 years. The experiment was conducted from May to July during the years 2018, 2020, and 2021. We conducted experiments in the two phases of the breeding season. A total of 45 nests were in the egg‐laying phase (i.e., uncompleted clutch) and 43 nests were in the incubation phase of nesting (completed clutch), which means we examined 88 nests in total. Our long‐term monitoring (2014–2022) of part of our focal population showed extremely low breeding fidelity. We, therefore, treated all tested shrike pairs as independent.

### 2.2 Study species

The red‐backed shrike is a mostly insectivorous passerine bird, but despite its medium size, it is also able to hunt small vertebrates (Cramp et al., 1994; Lefranc & Worfolk, 1997). The red‐backed shrike is a migratory species, which migrates to tropical Africa during the autumn. It arrives at its breeding sites during May and starts nest building (Morelli, 2012), choosing semi‐open habitat with scattered shrubs. It prefers shrubs with spikes and thorns, especially species like the wild rose (*Rosa canina*), blackthorn (*Prunus spinosa*), or hawthorn (*Crataegus* spp.) (Olsson, 1995).

The red‐backed shrike is strictly territorial in the breeding season. The open nest is built by both sexes, but especially by males, who also choose the nest site. The first clutch contains three to seven eggs. It can also have a replacement clutch in a new nest. The incubation lasts on average for 14–16 days. After hatching, the young stay in the nest on average for 14–16 days. After 25 days, the young are able to hunt some insects, and in approx. 42 days become independent (Lefranc & Worfolk, 1997; Lovászi & Moskát, 2004).

Both shrike parents defend their nest against intruders vigorously (Strnad et al., 2012; Tryjanowski & Goławski, 2004). During our long‐term research on shrikes, we scarcely observed any interterritorial interferences when defending their nests. The neighbors usually do not participate in defense on each other nests. The red‐backed shrike is a strongly territorial species, which does not have many possibilities of interaction except male and female within the pair or the interactions between parents and their fledglings. The minimum distance between shrikes' nests at our study locality was 51 m during the last 10 years of research.

### 2.3 Experimental design

**FIGURE 1:**
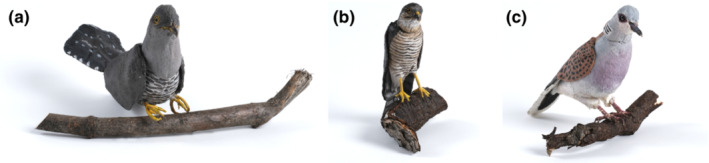
Textile dummies presented in the experiments. (a) Cuckoo (*Cuculus canorus*), (b) Eurasian sparrowhawk (*Accipiter nisus*), and (c) Turtle dove (*Streptopelia turtur*) common (photo by Kamila Horáková).

The dummies of intruders were presented as perching in an upright position on a 1.5 m high pole and c. 1 m far from the nest. All dummies had their wings folded and faced the nest. In this study, we used artificial textile dummies, which have previously been successfully used in several antipredator experiments (Antonová et al., 2021; Beránková et al., 2015; Němec et al., 2015, 2021; Nováková et al., 2020; Veselý et al., 2016; Figure 1). We opted for textile dummies as shrikes vigorously attack them and another approach, e.g., 3D‐printed plastic dummies (as used by Chen et al., 2022), could harm the tested birds. For each nest, GPS coordinates and the number of eggs were recorded, and we determined the phase of nesting, using the clutch size, egg temperature, and a floating test. As we conducted the experiments at the beginning of the nesting period, when parents are sensitive to disturbances, we presented only one dummy at each nest to reduce the time spent by the experimenters in the immediate surroundings of the nest. Before the start of each trial, we observed the visit rate of a potential territory by adults for up to 30 min to make sure the associated nest was active. If we did not record shrike parents in the vicinity of the nest during this period, we did not conduct the trial. After a positive recording of nest activity, we started the given trial, placing one of the dummies near the nest. The dummies were covered by cloth to prevent any early reaction of birds before and during installation. Each trial lasted for 10 min, and the beginning of each trial was the moment when at least one of the parents noticed the dummy. If neither of the parents noticed the dummy within this time, the trial was terminated and included in the dataset as a zero reaction. The reaction of the shrike was recorded on a HDC‐SD80 video camera accompanied by a detailed description of the behavior by a human observer. The video camera and the observer were located approx. 50 m from the nest to prevent any reaction to the observer. An acoustic recording was made on an Olympus WS 852 voice recorder, which was hidden under the presented dummy.

### 2.4 Dummies

We presented the following dummies at the shrikes' nests (see Figure 1 for photographs of actual dummies). (1) A female common cuckoo with gray upper parts, and a white belly with an undulating dark pattern. (2) A male Eurasian sparrowhawk with rufous underparts, a white belly with an undulating dark pattern, and brown‐gray upper parts. (3) As a baseline stimulus, we chose the turtle dove, which regularly occurs in our study area. It is comparable in size to the cuckoo and the sparrowhawk (Davies & Welbergen, 2008; Payne et al., 2005) and represents no threat to shrike adults or nest contents.

### 2.5 Statistical analyses

For statistical analyses, we used four behavioral data types. Two were in binomial form: the occurrence of at least one attack (flight toward the dummy, which may end with physical contact with the dummy) and the occurrence of at least one alarm call (the so‐called “chack” call—Ash, 1970; Harris & Franklin, 2000). We further recorded the number of flyovers and flights above the dummy (but not directed toward the dummy) as a measure of guarding the dummy. Lastly, we analyzed the number of attacks performed in experiments where at least one attack occurred. All behaviors were scored together for both parents. In most examples, only one parent (the male) was active.

To evaluate the effect of the predictor variables on both binomially scored responses (attack and alarm occurrence), we used the generalized linear model (GLM, binomial error distribution and logit link, command glm in R 4.1.1). We tested for the effect of two predictors and one interaction of predictors: dummy, nesting stage × dummy, and number of eggs. We opted for the interaction of the nesting stage and dummy type, as we predicted a different effect for nesting stage on responses to particular dummies. We compared the null model with the subsequent models using a likelihood ratio test following binomial distribution (Chi‐squared test). To compare particular dummies, we used a Fisher LSD post hoc test with correction for repeated comparisons.

To evaluate the effect of predictors on the number of flyovers and the number of attacks performed in the experiments (following Gaussian distribution), where at least one attack occurred, we used linear models (LM), command lm in R 4.1.1. We tested for the effect of two predictors and one interaction of predictors: dummy, nesting stage × dummy, and number of eggs. We compared the null model with the subsequent models using a likelihood ratio test following Gaussian distribution (*F* test). To compare particular dummies, we used a Tukey HSD post hoc test with Tukey correction for repeated comparisons.

## 3 RESULTS

**FIGURE 2:**
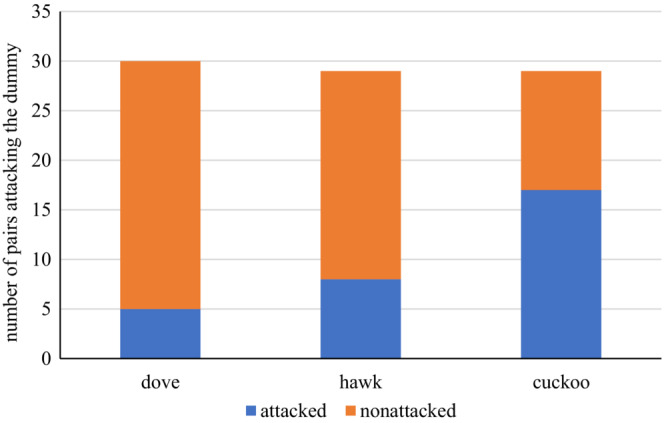
Number of experiments, in which at least one attack on the presented dummy occurred. Dove—turtle dove (*Streptopelia turtur*), hawk—Eurasian sparrowhawk (*Accipiter nisus*), and cuckoo—common cuckoo (*Cuculus canorus*).

The dummy type significantly affected the occurrence of attacks in the experiment, while the interaction of the phase of nesting and the dummy and clutch size did not (GLM, Table 1). Shrikes were significantly more willing to attack the cuckoo than either the dove (Fisher LSD post hoc test, *z* = 3.167, *p* = .004; Figure 2) or the sparrowhawk (Fisher LSD post hoc test, *z* = 3.341, *p* = .040; Figure 2). The sparrowhawk was attacked in an equal number of experiments to the dove (Fisher LSD post hoc test, *z* = 1.003, *p* = .574; Figure 2).

**TABLE 1:** Effect of predictors on particular behavioral responses of shrikes.

Response	Predictor	df	Chi/*F*	*p*
Attack occurrence	**Dummy**	**2**	**12.296**	**.002**
	Nesting phase × dummy	3	1.844	.834
	Number of eggs	1	0.128	.721
Number of attacks	**Dummy**	**2**	**2.886**	**.017**
	Nesting phase × dummy	3	1.863	.361
	Number of eggs	1	0.324	.574
Number of flyovers	Dummy	2	2.311	.105
	Nesting phase × dummy	3	1.795	.375
	Number of eggs	1	0.162	.688
Alarm occurrence	**Dummy**	**2**	**6.807**	**.033**
	Nesting phase × dummy	3	1.079	.779
	Number of eggs	1	0.160	.687

*Note*: Significant effect in bold. × Indicates interaction of factors.

**FIGURE 3:**
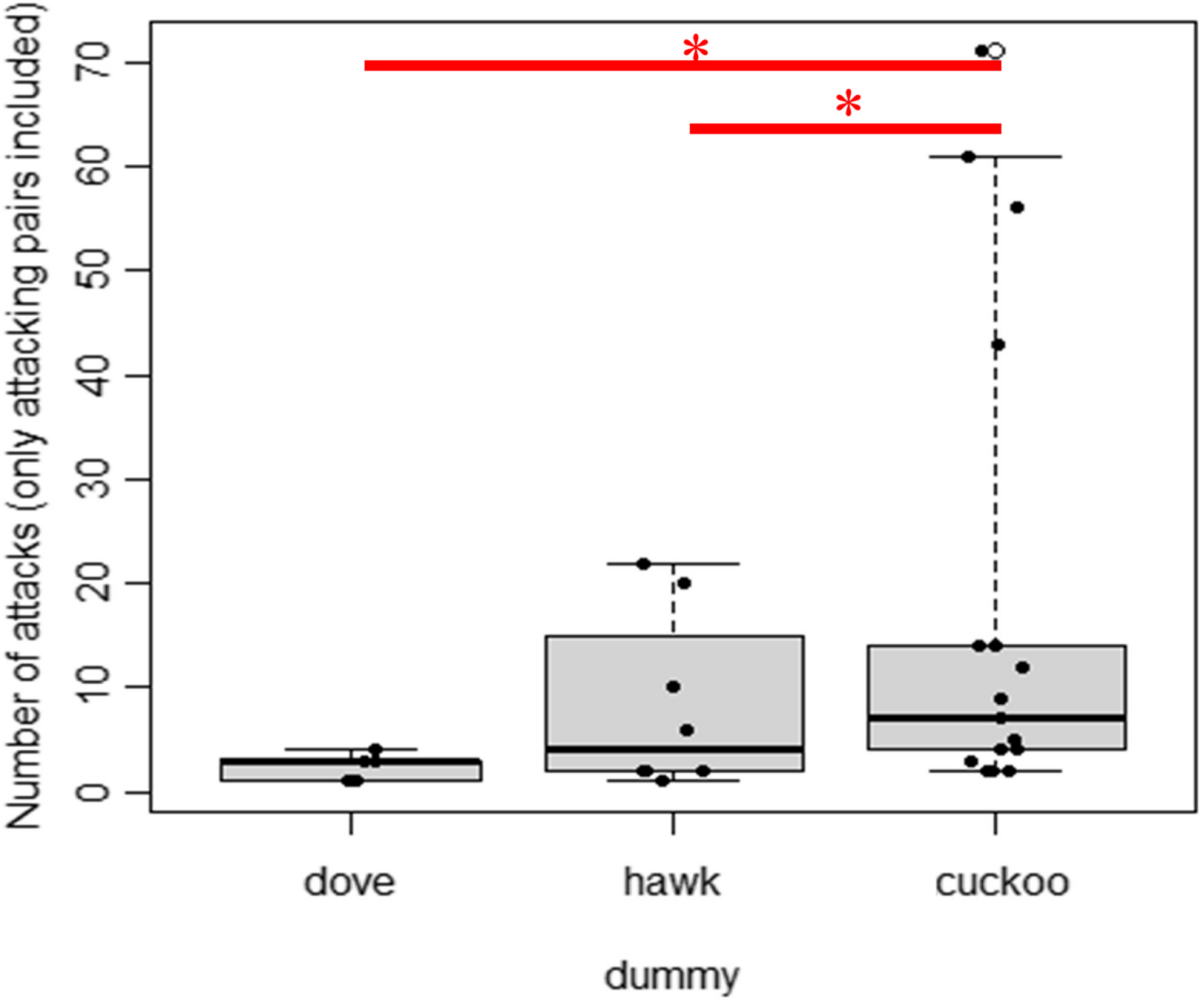
Number of attacks performed toward particular dummies in experiments, where at least one attack occurred. Dove—turtle dove (*Streptopelia turtur*), hawk—Eurasian sparrowhawk (*Accipiter nisus*), and cuckoo—common cuckoo (*Cuculus canorus*). The horizontal line within the box is median, asterisk indicates mean, boxes cover 75% of observations, whiskers cover 95% of observations, black dots represent the distribution of the observations, white dots represent the outliers, and significant differences are highlighted by a red line and asterisk.

The number of alarm attacks performed by shrikes in experiments where at least one attack occurred was significantly affected by the type of presented dummy, and the effects of the interaction of the nesting phase and dummy type and clutch size were not significant (LM, Table 1). Shrikes attacked the cuckoo most vigorously, significantly more than the dove (Tukey HSD post hoc test, *t* = 2.726, *p* = .021; Figure 3) and the sparrowhawk (Tukey HSD post hoc test, *t* = 2.324, *p* = .039; Figure 3). The sparrowhawk was attacked slightly more often than the dove (Tukey HSD post hoc test, *t* = 1.545, *p* = .084; Figure 3).

**FIGURE 4:**
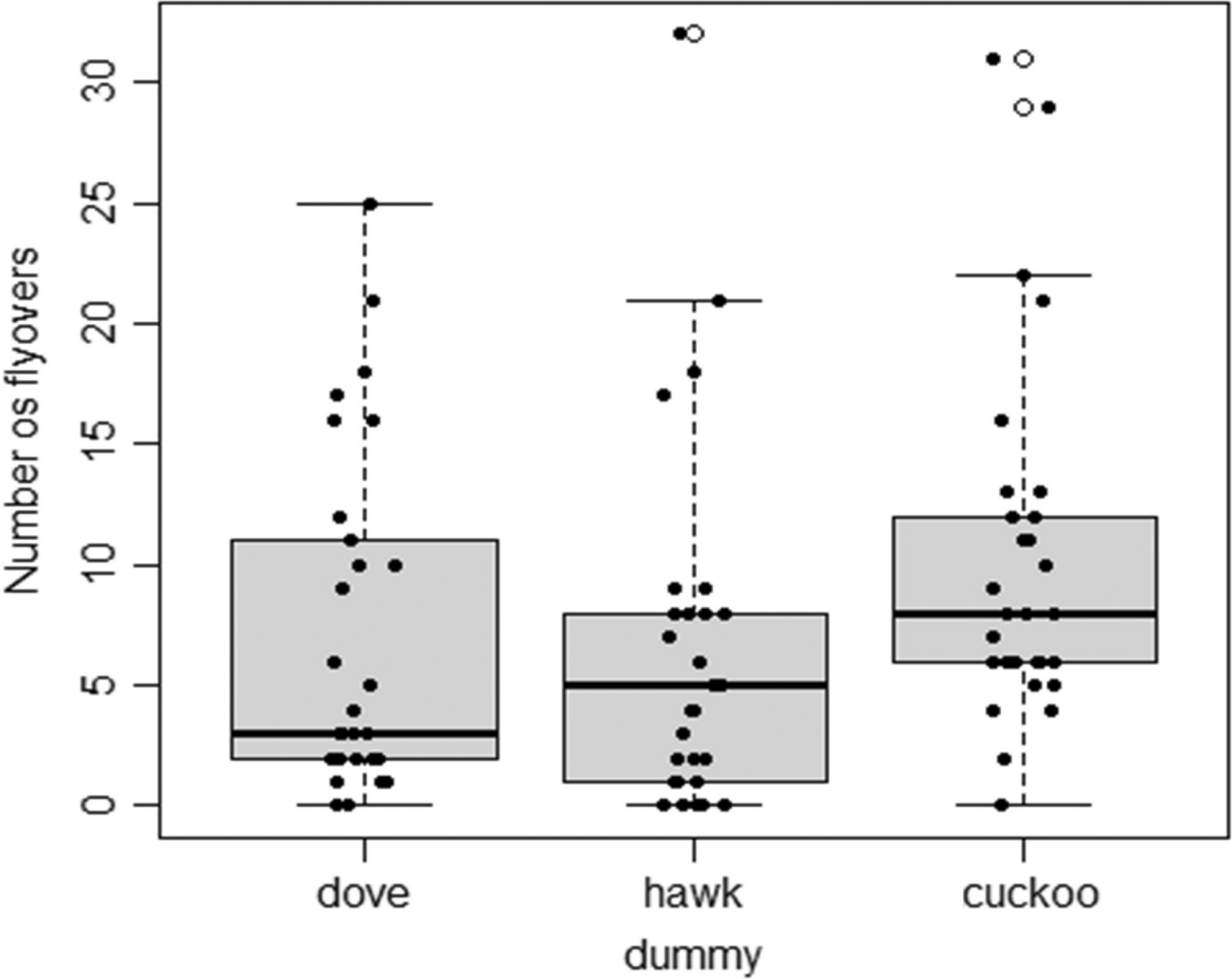
Number of flyovers performed toward particular dummies in all experiments. Dove—turtle dove (*Streptopelia turtur*), hawk—Eurasian sparrowhawk (*Accipiter nisus*), cuckoo—common cuckoo (*Cuculus canorus*). The horizontal line within the box is median, asterisk indicates mean, boxes cover 75% of observations, whiskers cover 95% of observation, dots represent outliers, black dots represent the distribution of the observations, and white dots represent the outliers.

The number of flyovers was not affected by any of the three selected predictors (LM, Table 1). Shrikes performed inspection flights in the presence of all dummies equally often (Figure 4).

**FIGURE 5:**
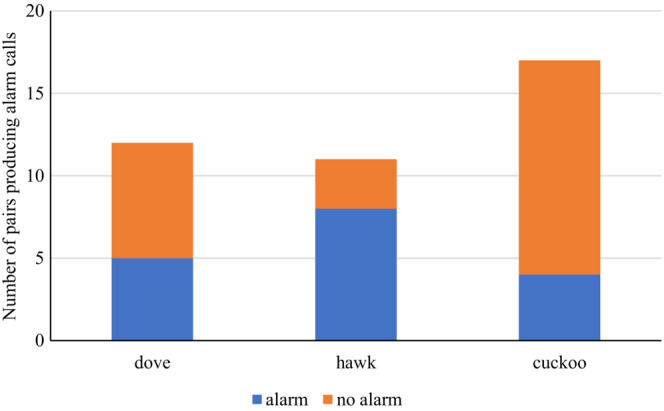
Number of experiments in which at least one alarm call was given. Dove—turtle dove (*Streptopelia turtur*), hawk—Eurasian sparrowhawk (*Accipiter nisus*), cuckoo—common cuckoo (*Cuculus canorus*).

The dummy type significantly affected the occurrence of alarm calls in the experiment, while the interaction of the phase of nesting and the dummy and clutch size did not (GLM, Table 1). Shrikes were more willing to produce alarm calls in the presence of the sparrowhawk dummy than in the presence of the cuckoo (Fisher LSD post hoc test, *z* = 2.437, *p* = .039; Figure 5). In the presence of the dove, the shrikes produced alarm calls equally often as in the presence of the sparrowhawk (Fisher LSD post hoc test, *z* = 1.472, *p* = .304; Figure 5) as well as the cuckoo (Fisher LSD post hoc test, *z* = 1.029, *p* = .558; Figure 5).

## 4 DISCUSSION

We found a significant difference between the reaction of the red‐backed shrikes to the presence of the common cuckoo (brood parasite) and the Eurasian sparrowhawk (predator) at their nests. In the presence of the cuckoo, the shrikes reacted very aggressively, commonly using attacks, usually many within a short time, while they did not produce many “chack” alarm calls. They commonly produce calls associated with contact attacks (physically striking with beak or claw) on the dummy (Ash, 1970; Harris & Franklin, 2000). In the presence of the sparrowhawk, shrikes tended to produce alarm calls and performed only inspection flights over the dummy. These results confirm that shrikes are able to distinguish between the common cuckoo and the Eurasian sparrowhawk and that cuckoo–hawk mimicry is not effective in the case of red‐backed shrikes (Davies, 2015).

In the presence of the sparrowhawk dummy, shrikes used alarm calls and only guarded the dummy, rarely attacking it. This suggests that shrikes fear the sparrowhawk and avoid attacking it directly. This concurs with our previous studies (Strnad et al., 2012; Strnadová et al., 2018) showing that shrikes are able to assess the threat particular species represent and suppress their vigorous nest defense behavior if it would threaten the parents themselves. This also agrees with the study of Roncalli et al. (2019) showing trade‐offs between antipredatory and antiparasitics strategies. Welbergen and Davies (2008) showed that reed warblers also responded to the presence of cuckoos with graded alarm calls, while in the presence of the sparrowhawk they remained at a greater distance from the nest.

Our results are in accordance with the previous studies (Thorogood & Davies, 2012; Trnka & Grim, 2014; Trnka & Prokop, 2012) which tested the effect of cuckoo–hawk mimicry as a protection of the cuckoo against an aggressive host, the great reed warbler, or the reed warbler. In this study, the very same three dummies as in our experiments were presented near the warbler nests. Great reed warblers were able to distinguish between the presented dummies and reacted differently to them. The warblers did attack either the cuckoo or the sparrowhawk, but the reaction to the cuckoo was significantly more aggressive and frequent. The authors conclude that the cuckoo–hawk mimicry was thereby disputed. Similarly, Welbergen and Davies (2008) showed that reed warblers also displayed a higher level of antipredation behavior in the presence of a cuckoo than in the presence of a sparrowhawk, which suggests the low efficacy of cuckoo–hawk mimicry regarding this species. On the contrary, Davies and Welbergen (2008) observed the attendance of great tits and blue tits at the feeder in the presence of four dummies—the common cuckoo, Eurasian sparrowhawk, and harmless controls—collared dove (*Streptopelia decaocto*) or teal (*Anas crecca*). The authors found no difference in attendance to feeders in the presence of a cuckoo and a sparrowhawk (attendance was zero in both cases), which suggests that tits are not able to differentiate between cuckoos and sparrowhawks. The most likely explanation of this difference is that tits are not common hosts of cuckoos as they commonly breed in tree hollows inaccessible to cuckoos (Yu et al., 2017). The evolutionary pressure to distinguish between cuckoos and sparrowhawks may thus not be as strong as in the case of warblers or shrikes, common cuckoo hosts.

A previous study suggested that physical attacks toward adult cuckoos may result in lower parasitation by cuckoos (Dyrcz & Hałupka, 2006). The question remains of whether the intensity of shrike attacks toward the cuckoo is so high that it could cause a decrease in parasitation by cuckoos. We may see some measure of aggressivity in the nest defense behavior of shrikes if we compare our results with our previous study where they were confronted with several species of predators (Strnadová et al., 2018). In the previous study, only 25% of shrikes attacked the Eurasian jay (*Garrulus glandarius*), a common nest predator. Two raptors, the Eurasian sparrowhawk and common kestrel (*Falco tinnunculus*), were attacked even more scarcely (around 15% of shrikes). Almost 60% of shrikes attacked the cuckoo in our recent experiments, which is really an intensive aggression level. We may thus imagine that this behavior is at least part of the reason why cuckoos have abandoned red‐backed shrikes as potential hosts.

The reaction of shrikes to the presented dummies was not affected by the phase of nesting. This result is surprising because the cuckoo prefers to parasite its hosts in the egg‐laying phase rather than the incubating phase to increase the likelihood of acceptance of the egg (Davies, 2000). Based on this theory, host species should react to the presence of a cuckoo more in the egg‐laying phase. On the other hand, as the cuckoo can depredate the whole clutch and force the host to rebreed, it represents a threat during the whole nesting season (Davies, 2011), even in the nestling phase (Šulc et al., 2020). Moreover, it has been documented that most host species react very aggressively toward adult cuckoos during the whole nesting season (Jelínek et al., 2021).

In addition, the response of shrikes was not affected by clutch size, although a higher number of eggs represents higher investments in the brood and therefore the nest defense should be stronger with regard to larger clutches (Redondo, 1989; Wiklund & Andersson, 1994). However, there are also studies showing no effect on clutch size (Curio et al., 1984; Lazarus & Inglis, 1986; Curio, 1987). In our data, small clutch size means not only low parental investment but also incomplete clutches, which could also weaken the effect of clutch size in our data.

## 5 CONCLUSIONS

Our results show that shrikes are not fooled by cuckoo–hawk mimicry, can differentiate these two species, and attack cuckoos vigorously. This high level of aggression may be a reason why the common cuckoo has abandoned the shrike as a potential host, as the adults were not able to successfully parasitize shrike nests. At our study locations, the shrike population has not been parasitized by cuckoos for at least 20 years, but the shrikes obviously treat the adult cuckoos as a threat to their nests and spend a lot of energy chasing them away. It is very likely that there are more reasons why cuckoos abandoned shrikes as potential hosts. One of them can be the ability of the shrike to recognize the difference between parasitic and its own eggs, which was suggested in Hungarian populations, another could be low population densities of shrikes. Corroborating the importance of these factors needs further research.

## AUTHOR CONTRIBUTIONS

**Ladislava Krausová:** Data curation (lead); formal analysis (supporting); investigation (supporting); writing – original draft (lead). **Petr Veselý:** Formal analysis (equal); methodology (equal); supervision (supporting); writing – original draft (equal); writing – review and editing (lead). **Michaela Syrová:** Data curation (supporting); formal analysis (equal); methodology (equal); supervision (supporting); writing – original draft (equal); writing – review and editing (equal). **Kateřina Antonová:** Data curation (supporting); writing – review and editing (supporting). **Ondřej Fišer:** Data curation (supporting); writing – review and editing (supporting). **Vanda Chlumská:** Data curation (supporting). **Markéta Pátková:** Data curation (supporting). **Šimon Pužej:** Data curation (supporting). **Roman Fuchs:** Methodology (supporting); supervision (lead); writing – review and editing (equal).

## FUNDING INFORMATION

This study was supported by the University of South Bohemia (048/2019/P).

## CONFLICT OF INTEREST

Authors declare no conflict of interest.

## Data Availability

The data that support the findings of this study will be openly available in Dryad.
